# Comparative associations of three nutritional indices with hematoma expansion after intracerebral hemorrhage

**DOI:** 10.3389/fnut.2026.1803984

**Published:** 2026-03-24

**Authors:** Shuang Zhao, Lin Zhang, Qinyu Zhou, Yun Lu, Yang Liu

**Affiliations:** 1 School of Clinical Medicine, Chengdu University of Traditional Chinese Medicine, Chengdu, Sichuan, China; 2 Department of Emergency Medicine, Hospital of Chengdu University of Traditional Chinese Medicine, Chengdu, Sichuan, China; 3 The Third Hospital of Mianyang, Sichuan Mental Health Center, Mianyang, Sichuan, China

**Keywords:** CONUT, hematoma expansion, intracerebral hemorrhage, PNI, TCBI

## Abstract

**Background:**

Hematoma expansion (HE) is a major determinant of early neurological deterioration after intracerebral hemorrhage (ICH) and remains a key target for early risk assessment. Nutritional indices obtained at hospital admission can reflect acute physiological vulnerability during hemorrhagic injury, but their relative relevance to HE has not been well established. This study investigated the associations between three commonly used nutritional indices and HE in patients with ICH.

**Methods:**

We retrospectively included patients with ICH admitted between January 2023 and December 2024. Nutritional status at admission was assessed using the Prognostic Nutritional Index (PNI), the Triglycerides × Total Cholesterol × Body Weight Index (TCBI), and the Controlling Nutritional Status (CONUT) score. HE was defined as a relative hematoma growth >33% or an absolute increase >6 mL on follow-up computed tomography. Associations between each nutritional index and HE were examined using univariable and multivariable logistic regression. Restricted cubic spline (RCS) analysis was used to characterize the association pattern, and subgroup and sensitivity analyses were performed to evaluate consistency of the findings.

**Results:**

Among 349 included patients, 42 (12.0%) developed HE. Higher CONUT scores were significantly associated with an increased risk of HE (OR = 1.29, 95%CI: 1.03–1.59, *p* = 0.02). In contrast, neither PNI nor TCBI demonstrated significant associations after multivariable adjustment. RCS analyses indicated a significant overall relationship between CONUT and HE without evidence of nonlinearity. This association remained consistent across predefined subgroups with no significant interactions and was confirmed in sensitivity analyses.

**Conclusion:**

This study demonstrated a significant association between nutritional status and HE in ICH. Among the nutritional indices examined, CONUT showed a positive association, suggesting its role as a practical nutritional risk indicator for early risk stratification of HE in ICH.

## 1 Introduction

Intracerebral hemorrhage (ICH) accounts for approximately 25% of all strokes and is associated with high mortality and poor functional outcomes compared with other stroke subtypes (1, 2). Hematoma expansion (HE) occurs in about 30% of ICH patients (3). It has been consistently associated with early neurological deterioration and adverse clinical outcomes, including increased mortality and prolonged hospitalization (4). Consequently, HE represents a critical early pathophysiological process and a key target for risk stratification in ICH.

The development of HE reflects a dynamic process rather than the initial bleeding event alone. Following vessel rupture, hematoma growth depends on the interplay between ongoing bleeding, local hemostatic capacity, and vascular stability (5, 6). These processes occur predominantly during the acute phase and are influenced not only by hemorrhagic lesion features but also by the patient’s baseline physiological and metabolic condition (7). In this context, nutritional status represents an important systemic factor. It reflects protein availability, lipid reserves, and overall metabolic support, all of which are integral to maintaining vascular integrity and coagulation balance during acute injury (8).

Malnutrition or nutritional risk is prevalent among patients with ICH and is associated with increased mortality, infectious complications, and unfavorable functional outcomes (9). Accordingly, objective nutritional indices derived from routinely available laboratory parameters have gained increasing attention in cerebrovascular research (10). The Prognostic Nutritional Index (PNI), derived from serum albumin and lymphocyte count, primarily reflects protein reserve and immune status (11). The Controlling Nutritional Status (CONUT) score integrates serum albumin, lymphocyte count, and total cholesterol and was originally developed as an in-hospital nutritional risk screening tool. In contrast to indices reflecting long-term nutritional reserve, CONUT is considered to capture acute nutritional-metabolic vulnerability at admission (12). The Triglycerides × Total Cholesterol × Body Weight Index (TCBI) is a lipid-based indicator reflecting energy reserves and has shown prognostic relevance in stroke populations (13–15). Nevertheless, comparative evidence evaluating PNI, CONUT, and TCBI specifically in relation to HE in ICH remains limited.

Therefore, this study aimed to evaluate the associations between three nutritional indices (PNI, TCBI, and CONUT) and HE in a retrospective cohort of patients with ICH. By comparing their relative performance in identifying HE risk, this study sought to clarify the potential role of nutritional status in early HE risk stratification and to provide evidence supporting the application of nutritional assessment in ICH care.

## 2 Materials and methods

### 2.1 Study population

**Figure 1:**
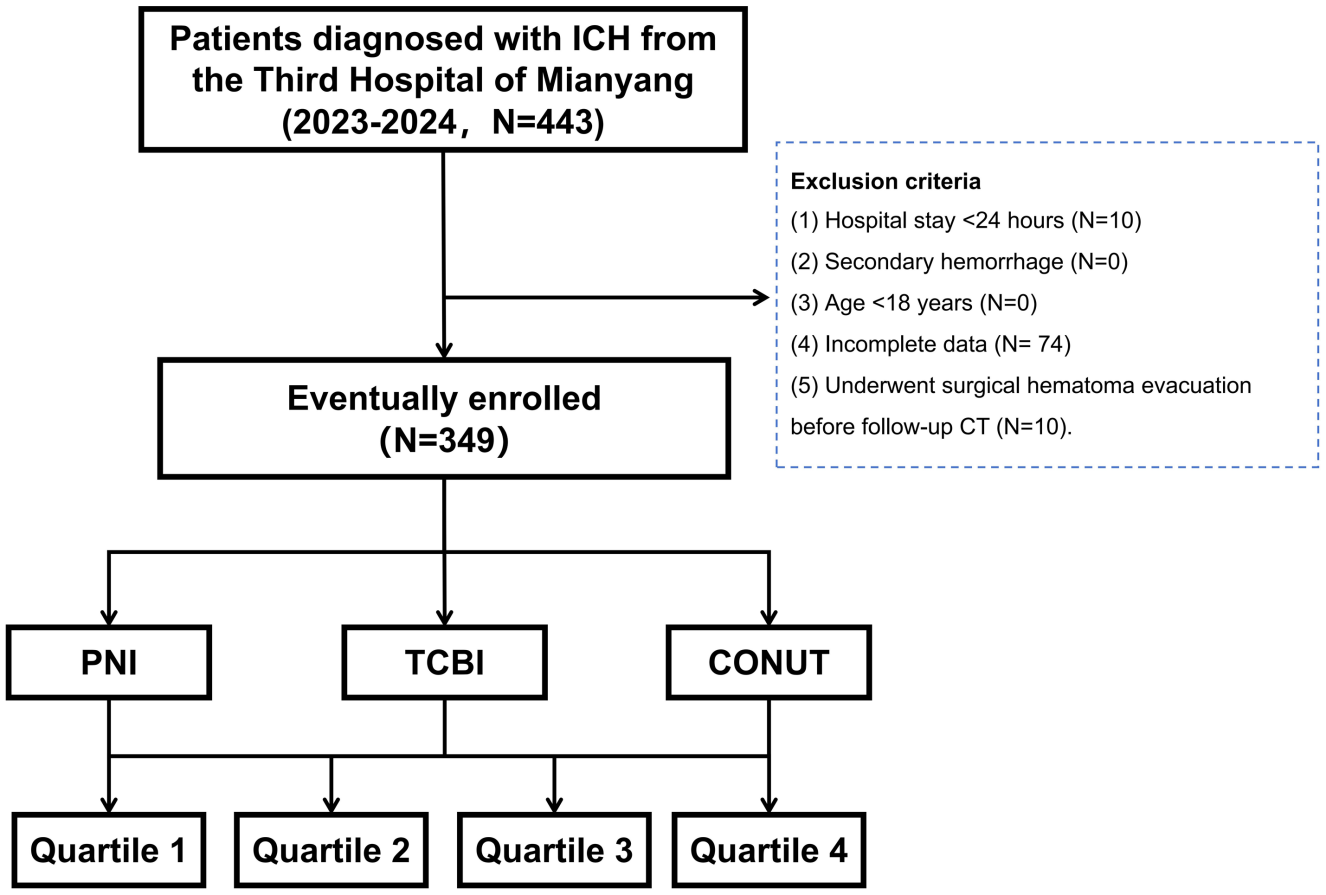
Flowchart of patient selection. ICH, intracerebral hemorrhage; TCBI, triglyceride-total cholesterol-body weight index; PNI, prognostic nutritional index; CONUT, controlling nutritional status.

This retrospective study was conducted between January 2023 and December 2024 at the department of Neurosurgery, the Third People’s Hospital of Mianyang. The study was approved by the institutional ethics committee, and informed consent was waived due to data de-identification (Ethical Approval No.: 2025–030-3). The overall study design and patient selection process are summarized in Figure 1.

### 2.2 Inclusion and exclusion criteria

Consecutive patients with primary ICH admitted between January 2023 and December 2024 were considered for inclusion. The diagnosis of ICH was initially confirmed by brain computed tomography (CT) at admission. To ensure data completeness and clinical consistency, we also applied the following exclusion criteria: (1) hospital stay shorter than 24 h; (2) traumatic brain injury, subarachnoid hemorrhage, brain tumors, and hemorrhagic transformation secondary to other conditions; (3) age younger than 18 years; and (4) patients with incomplete biochemical or imaging data relevant to this study; (5) patients who underwent surgical hematoma evacuation before follow-up CT.

### 2.3 Data collection and outcomes

Baseline demographic characteristics (age and gender) and clinical characteristics, including height, weight, smoking history, alcohol use, onset-to-admission time, and prior use of anticoagulant therapy were recorded. Clinical status at admission was evaluated using the Glasgow Coma Scale (GCS) score. Comorbidities, including hypertension, intraventricular hemorrhage, diabetes, hyperlipidemia, atrial fibrillation, and chronic obstructive pulmonary disease (COPD), were documented. All laboratory test results were obtained at the time of hospital admission as the first measurements for each patient, minimizing the influence of early interventions or acute treatment. For calculation of the nutritional indices according to the original published formulas, albumin, total cholesterol, and triglycerides were converted to the units required by the formulas (albumin from g/L to g/dL; total cholesterol from mmol/L to mg/dL; triglycerides from mmol/L to mg/dL). Nutritional status was assessed using three indices: PNI, TCBI, and CONUT. PNI was calculated as 10 × serum albumin(g/dL) + 5 × total lymphocyte count (10^9^/L) (16). TCBI was calculated as serum triglycerides (mg/dL) × total cholesterol (mg/dL) × body weight (kg) / 1,000 (17). The CONUT score was calculated according to the established scoring system based on serum albumin concentration, total lymphocyte count, and total cholesterol level, as previously described (18). The grading criteria for the CONUT score are presented in Supplementary Table S1. Nutritional indices were analyzed both as continuous variables and as quartiles.

All key variables required for this study were complete after applying the inclusion and exclusion criteria, ensuring no missing data for the primary analyses. For other variables, those with more than 10% missing values were excluded, while variables with 10% or less missing data were handled using multiple imputation to minimize bias and preserve statistical efficiency.

The primary outcome was HE, defined as a relative hematoma growth >33% or an absolute increase >6 mL on follow-up CT compared with the baseline scan. Follow-up CT was performed within 24 h after admission (19). Baseline and follow-up CT examinations were obtained at our institution as routine non-contrast head CT, with official radiology reports issued by the Department of Radiology. The radiology reports documented the hematoma diameters (A, B, and C). Hematoma volume was calculated by the investigators using the A × B × C/2 method based on the reported A, B, and C values (20).

### 2.4 Statistical analysis

The normality of continuous variables was assessed using the Shapiro–Wilk test. Normally distributed variables were summarized as means with standard deviations (SD) and compared using independent t-tests. Non-normally distributed variables were expressed as medians with interquartile ranges (IQR) and analyzed using the Mann–Whitney U test. Categorical variables were reported as frequencies and proportions and compared using the chi-square test or Fisher’s exact test, as appropriate.

The distributions of nutritional indices were visualized using histograms and violin plots overlaid with boxplots. Each nutritional index was analyzed both as a categorical variable (quartiles) and as a continuous variable. Quartiles of each nutritional index were defined based on the distribution in the overall study population. Univariable and multivariable logistic regression analyses were used to examine the associations between nutritional indices and HE. Covariates were selected based on demographic characteristics and previous studies, including age, gender, hematoma location, onset-to-admission time, baseline hematoma volume, intraventricular hemorrhage (IVH), GCS and systolic blood pressure (SBP) (21). Three models were constructed: a crude model; Model 1 adjusted for age and gender. Model 2 further adjusted for hematoma location, onset-to-admission time, baseline hematoma volume, IVH, GCS and SBP.

Restricted cubic spline analyses were performed to explore potential non-linear relationships between nutritional indices and HE. Subgroup analyses were conducted using adjusted multivariable models across predefined strata of age, gender, hemorrhage location and hypertension. Interaction terms were tested to assess potential effect modification.

Given that HE predominantly occurs within the first 24 h after symptom onset, sensitivity analyses were restricted to patients admitted within 24 h after symptom onset to assess the robustness of the findings. In addition, for any composite nutritional index showing a statistically significant association with HE in the primary analyses, we performed component-substitution sensitivity analyses by replacing the composite index with its constituent components in otherwise identical multivariable models to assess the robustness of the association. Finally, for significant nutritional index in the primary analyses, we conducted a clinically anchored cut-off sensitivity analysis by re-categorizing the index according to clinically meaningful thresholds reported in prior literature. The re-categorized variable was then entered into the same multivariable models as in the primary analyses.

All statistical analyses were performed using R software (version 4.5.2). A two-sided *p* value < 0.05 was considered statistically significant.

## 3 Results

### 3.1 Baseline characteristics

Among the 349 included patients, 42 (12.03%) developed HE. Baseline characteristics are summarized in Table 1 and the characteristics of excluded patients are presented in Supplementary Table S2. Patients with HE had higher CONUT scores at admission (median 4 vs. 3, *p* = 0.03) and presented earlier after symptom onset (median 3 vs. 4 h, *p* = 0.002). In addition, the HE group exhibited lower platelet counts and prolonged activated partial thromboplastin time, suggesting reduced hemostatic stability at presentation. Other baseline demographic and clinical characteristics, including baseline hematoma volume, were broadly comparable between groups.

**Table 1:** Baseline characteristics of the patients.

Characteristic	Overall (*N* = 349)	Non-HE (*N* = 307)	HE (*N* = 42)	*P*
Age	66.00 (55.00,74.00)	66.00 (55.00, 74.00)	66.00 (54.00, 75.00)	0.96
Gender (male,%)	227 (65.04)	198 (64.50)	29 (69.05)	0.56
Weight (kg)	62.79 ± 0.62	63.09 ± 0.66	60.63 ± 1.86	0.2
Height (cm)	160.00 (155.00, 168.00)	160.00 (155.00, 168.00)	161.50 (156.00, 168.00)	0.62
Smoke (*n*,%)	117 (33.52)	98 (31.92)	19 (45.24)	0.09
Alcohol (*n*, %)	79 (22.64)	69 (22.48)	10 (23.81)	0.85
Use of anticoagulant (*n*, %)	14 (4.01)	10 (3.25)	4 (9.52)	0.07
Onset-to-admission time (h)	4.00 (3.00, 12.00)	4.00 (3.00, 15.00)	3.00 (2.00,5.00)	0.002
Scores				
GCS	13.00 (10.00, 15.00)	13.00 (10.00, 15.00)	13.00 (10.00, 15.00)	0.48
TCBI	992.13 (625.01, 1707.30)	1019.42 (633.86, 1775.52)	839.70 (490.06, 1308.65)	0.07
CONUT	3.00 (2.00, 4.00)	3.00 (2.00, 4.00)	4.00 (2.00, 5.00)	0.03
PNI	44.95 (41.80, 48.35)	45.00 (41.90, 48.60)	44.65 (41.25, 46.55)	0.19
Hematoma location (*n*, %)				0.42
Supratentorial	297 (85.10)	263 (85.67)	34 (80.95)	
Infratentorial	52 (14.90)	44 (14.33)	8 (19.05)	
Hematoma volume (ml)				0.89
Supratentorial	11.46 (4.48, 25.72)	11.46 (4.12, 26.12)	11.39(6.38, 16.64)	
Infratentorial	3.78 (1.56, 11.73)	3.78 (1.44, 11.73)	5.06 (2.28, 11.06)	
Comorbidities (*n*, %)				
IVH	141 (40.40)	126 (41.04)	15 (35.71)	0.51
Hypertension	298 (85.39)	263 (85.67)	35 (83.33)	0.68
Diabetes	49 (14.04)	45 (14.66)	4 (9.52)	0.36
Hyperlipidemia	69 (19.77)	63 (20.52)	6 (14.29)	0.34
Atrial fibrillation	13 (3.72)	11 (3.58)	2 (4.76)	0.71
COPD	39 (11.17)	34 (11.07)	5 (11.90)	0.87
Laboratory tests				
White blood cell (10^9^ /L)	8.80 (6.59, 11.20)	8.88 (6.59, 11.20)	7.96 (6.22, 11.21)	0.71
Red blood cell (10^9^ /L)	4.20 (3.83, 4.58)	4.20 (3.83, 4.59)	4.20 (3.99, 4.46)	0.87
Hemoglobin (g/dL)	128.50 (116.00, 140.00)	129.00 (116.00, 140.00)	126.00 (116.00, 137.00)	0.67
Lymphocyte count (10^9^ /L)	0.93 (0.66, 1.27)	0.94 (0.67, 1.29)	0.90 (0.60, 1.26)	0.12
Neutrophils (10^9^ /L)	7.09 (4.90, 9.50)	7.11 (4.91, 9.41)	6.31 (4.90, 10.22)	0.87
Platelet (10^9^ /L)	153.00 (113.50, 193.00)	155.00 (115.00, 198.00)	143.00 (99.00, 171.00)	0.04
INR	1.02 (0.97, 1.07)	1.01 (0.97, 1.07)	1.03 (0.99, 1.09)	0.32
Prothrombin time (s)	11.70 (11.20, 12.20)	11.70 (11.20,12.20)	11.90 (11.25, 12.40)	0.23
APTT (s)	26.20 (24.90, 28.20)	26.00 (24.80, 28.00)	27.55 (25.40, 29.30)	0.03
FDP (ug/ml)	5.10 (3.20, 10.10)	5.15 (3.20, 10.30)	4.70 (3.10, 7.90)	0.61
D-Dimer (ng/ml)	0.60 (0.33, 1.10)	0.57 (0.33, 1.10)	0.75 (0.34, 1.10)	0.71
Thrombin time (s)	17.80 (17.10, 18.40)	17.80 (17.10, 18.40)	17.65 (17.00, 18.10)	0.35
Fibrinogen (g/L)	2.94 (2.49, 3.62)	2.98 (2.50, 3.64)	2.80 (2.43, 3.27)	0.43
ALT (U/L)	19.00 (13.50, 29.00)	19.00 (14.00, 29.00)	21.00 (13.00, 29.00)	0.87
AST (U/L)	25.00 (20.00, 33.00)	25.00 (20.00, 34.00)	26.00 (20.00, 31.00)	0.67
Albumin (g/L)	39.70 (37.30, 42.60)	39.70 (37.30, 42.70)	39.50 (37.30, 42.00)	0.6
Triglycerides (mmol/l)	1.14 (0.80, 1.75)	1.14 (0.82, 1.78)	1.00 (0.62, 1.43)	0.06
Total cholesterol (mmol/l)	4.18 (3.69, 4.70)	4.18 (3.71, 4.70)	4.17 (3.35, 4.73)	0.46
Glucose (mmol/L)	6.27 (5.31, 7.67)	6.29 (5.31, 7.76)	6.13 (5.52, 7.57)	0.83
Sodium (mmol/L)	140.00 (138.00, 142.00)	140.00 (138.00, 142.00)	141.00 (139.00, 142.00)	0.52
Potassium (mmol/L)	3.76 (3.46, 4.02)	3.75 (3.45, 4.02)	3.83 (3.53, 4.07)	0.37
Calcium (mmol/L)	2.19 ± 0.12	2.19 ± 0.12	2.17 ± 0.13	0.29
Chloride (mmol/L)	105.90 (103.50, 108.30)	105.80 (103.60, 108.20)	106.00 (103.40, 109.10)	0.78
Creatinine (umol/L)	62.00 (50.50, 76.50)	63.00 (51.00, 78.00)	58.00 (50.00, 72.00)	0.23
Blood urea nitrogen (mmol/L)	5.21 (4.32, 6.63)	5.17 (4.32, 6.72)	5.27 (4.46, 5.89)	0.94
Uric acid (umol/L)	301.00 (227.50, 374.00)	301.00 (227.00, 377.00)	284.00 (237.00, 363.00)	0.78

Continuous variables were expressed as mean ± standard deviation (SD) for normally distributed variables, median.

GCS, glasgow coma scale; TCBI, triglyceride-total cholesterol-body weight index; PNI, prognostic nutritional index; CONUT, controlling nutritional status; IVH, intracerebral ventricular hemorrhage; COPD, chronic obstructive pulmonary disease; INR, international normalized ratio; APTT, activated partial thromboplastin time; FDP, fibrin degradation products; ALT, alanine aminotransferase; AST, aspartate aminotransferase.

**Figure 2:**
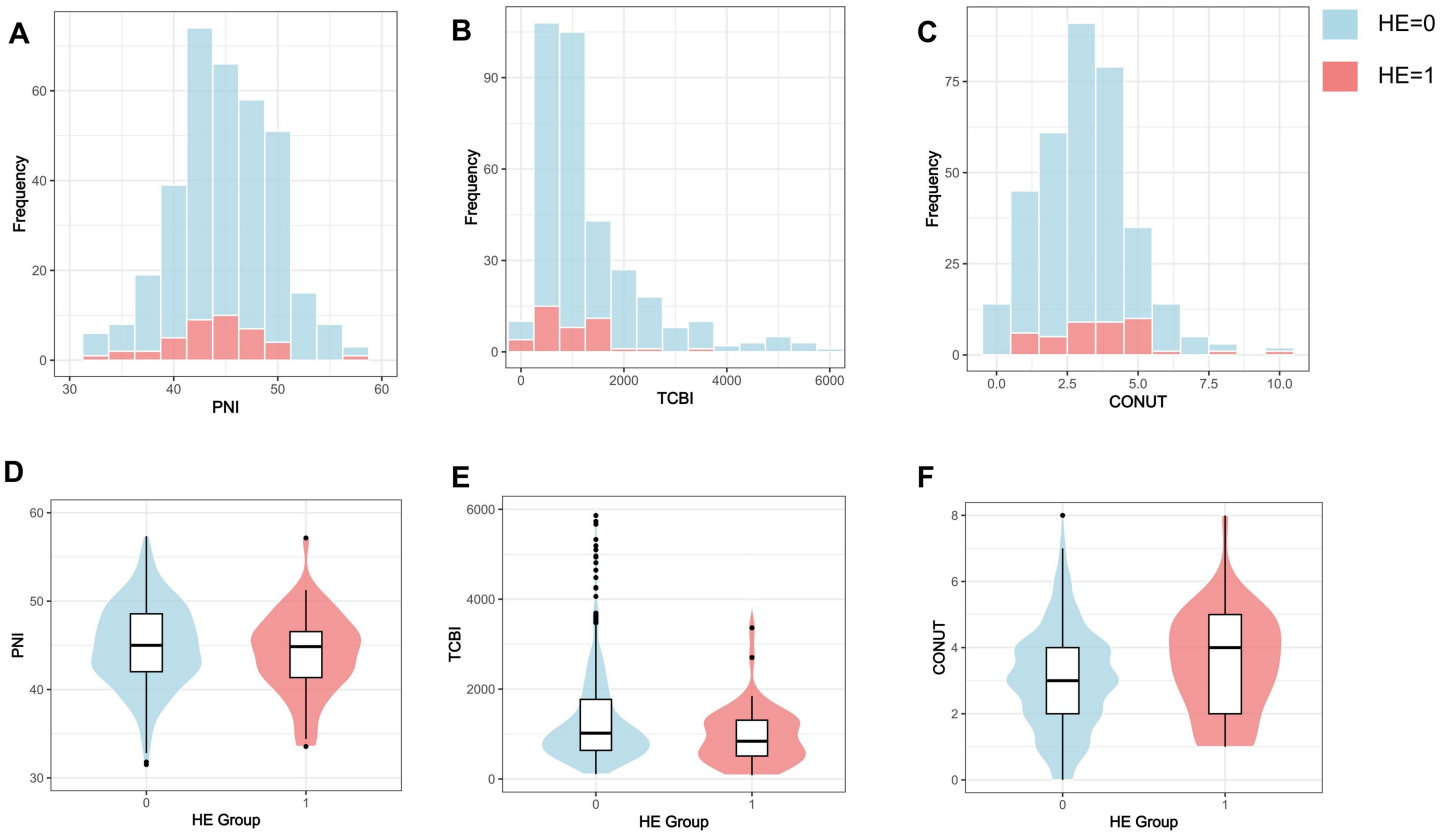
Distributions of nutritional indices according to HE status. **(A–C)** Frequency distribution histograms of PNI, TCBI, and CONUT score stratified by HE status. **(D–F)** Violin plots with embedded boxplots of PNI, TCBI, and CONUT according to HE status. HE, hematoma expansion; TCBI, triglyceride-total cholesterol-body weight index; PNI, prognostic nutritional index; CONUT, controlling nutritional status.

The distributions of the nutritional indices are shown in Figure 2. PNI demonstrated an approximately symmetric distribution centered in the mid-40s. TCBI showed a non-normal distribution with a pronounced right-skewed tail. CONUT scores displayed a discrete distribution concentrated in the lower-to-moderate range, with mild right skewness.

### 3.2 Association between nutritional indices and HE

Univariable and multivariable logistic regression analyses examining the associations between nutritional indices and HE are presented in Table 2. When analyzed as continuous variables, CONUT was associated with an increased risk of HE across all models (Model 2: OR 1.29, 95% CI 1.03–1.59, *p* = 0.02). In contrast, neither PNI nor TCBI showed a significant association with HE. Given the markedly right-skewed distribution of TCBI, its continuous effect was modeled and reported per 1-SD increase (standardized TCBI) to facilitate interpretation and reduce dependence on the original measurement scale.

**Table 2:** Univariable and multivariable logistic regression analyses.

Categories	Unadjusted model	*P*	Model I	*P*	Model II	*P*
	OR (95% CI)		OR (95% CI)		OR (95% CI)	
PNI						
Continuous	0.94 (0.89–1.01)	0.1	0.94 (0.88–1.00)	0.09	0.93 (0.86–1.00)	0.06
Quartile 1 (*N* = 88)	Reference		Reference		Reference	
Quartile 2 (*N* = 87)	0.66(0.26–1.64)	0.38	0.67 (0.27–1.69)	0.41	0.49 (0.18–1.31)	0.15
Quartile 3 (*N* = 87)	0.83 (0.35–1.98)	0.68	0.83 (0.34–2.01)	0.69	0.66 (0.25–1.73)	0.40
Quartile 4 (*N* = 87)	0.66 (0.26–1.64)	0.38	0.65 (0.24–1.71)	0.39	0.58 (0.20–1.69)	0.32
TCBI						
Continuous (per 1-SD increase)	0.88 (0.59–1.31)	0.54	0.88 (0.59–1.33)	0.56	0.92 (0.61–1.38)	0.67
Quartile 1 (*N* = 88)	Reference		Reference		Reference	
Quartile 2 (*N* = 87)	0.53 (0.21–1.34)	0.19	0.52 (0.21–1.32)	0.17	0.49 (0.19–1.28)	0.15
Quartile 3 (*N* = 87)	1.10 (0.49–2.44)	0.81	1.08 (0.47–2.46)	0.86	0.96 (0.46–2.25)	0.94
Quartile 4 (*N* = 87)	0.32 (0.11–0.93)	0.04	0.30 (0.09–0.92)	0.04	0.34 (0.21–1.75)	0.23
CONUT						
Continuous	1.21 (1.01–1.46)	0.03	1.21 (1.01–1.46)	0.03	1.29 (1.03–1.59)	0.02
Quartile 1 (*N* = 120)	Reference		Reference		Reference	
Quartile 2 (*N* = 91)	1.08 (0.43–2.74)	0.86	1.11 (0.43–2.83)	0.82	1.10 (0.43–2.84)	0.84
Quartile 3 (*N* = 79)	1.27 (0.50–3.23)	0.61	1.27 (0.49–3.29)	0.62	1.25 (0.46–3.37)	0.66
Quartile 4 (*N* = 59)	2.80 (1.16–6.71)	0.02	2.80 (1.15–6.82)	0.02	3.14 (1.23–8.05)	0.02

Model 1: adjusted for age and gender.

Model 2: adjusted for age, gender, hematoma location, onset-to-admission time, baseline hematoma volume, IVH, GCS and SBP.

For continuous analyses, TCBI is reported per 1-SD increase because its distribution is markedly right-skewed and the OR per 1-unit increase is scale-dependent and clinically unintuitive; PNI and CONUT are reported per 1-unit increase.

PNI: Quartile 1 (21.65–41.8), Quartile 2 (41.85–44.95), Quartile 3 (45–48.35), and Quartile 4 (48.45–57.35).

TCBI: Quartile 1 (85.08–625.01), Quartile 2 (627.26–992.13), Quartile 3 (998.69–1707.3), and Quartile 4 (1709.8–15922.48).

CONUT: Quartile 1 (0–2), Quartile 2 (3–3), Quartile 3 (4–4), and Quartile 4 (5–10).

OR, odds ratio; CI, confidence interval; TCBI, triglyceride-total cholesterol-body weight index; PNI, prognostic nutritional index; CONUT, controlling nutritional status; GCS, glasgow coma scale; SBP, systolic blood pressure.

Consistent with the continuous analysis, patients in the highest CONUT quartile had a significantly greater risk of HE compared with those in the lowest quartile. For TCBI, a lower risk was observed in the highest quartile in the unadjusted model and Model 1. However, this association was attenuated and no longer statistically significant after full adjustment. No significant associations were observed across PNI quartiles.

### 3.3 Restricted cubic spline analyses

**Figure 3:**
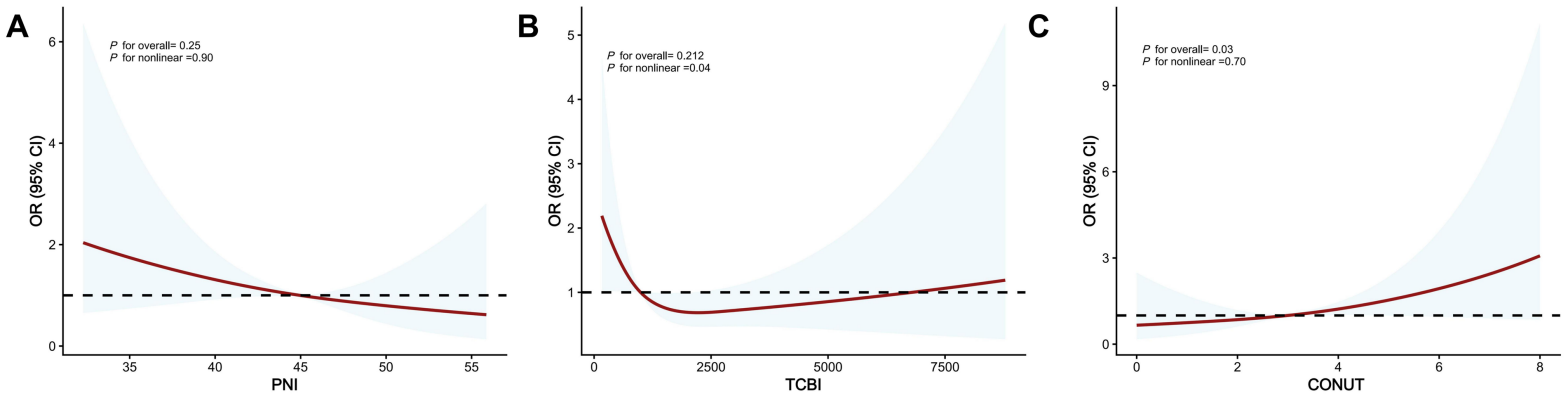
**A**–**C**: Restricted cubic spline curves showing the associations of PNI, TCBI, and CONUT with the risk of HE. OR, Odds Ratio; CI, Confidence Interval; CONUT, Controlling Nutritional Status; HE, Hematoma Expansion. TCBI, Triglyceride-Total Cholesterol-Body Weight Index; PNI, Prognostic Nutritional Index.

Restricted cubic spline analyses with three knots were performed to explore potential non-linear associations between nutritional indices and hematoma expansion (Figure 3). A significant overall association was observed for CONUT (P for overall associatio*n* = 0.03), with no evidence of non-linearity (P for non-linearity = 0.70), suggesting a relationship compatible with a linear trend. In contrast, no significant overall associations were detected for PNI or TCBI.

### 3.4 Subgroup and sensitivity analyses

**Figure 4:**
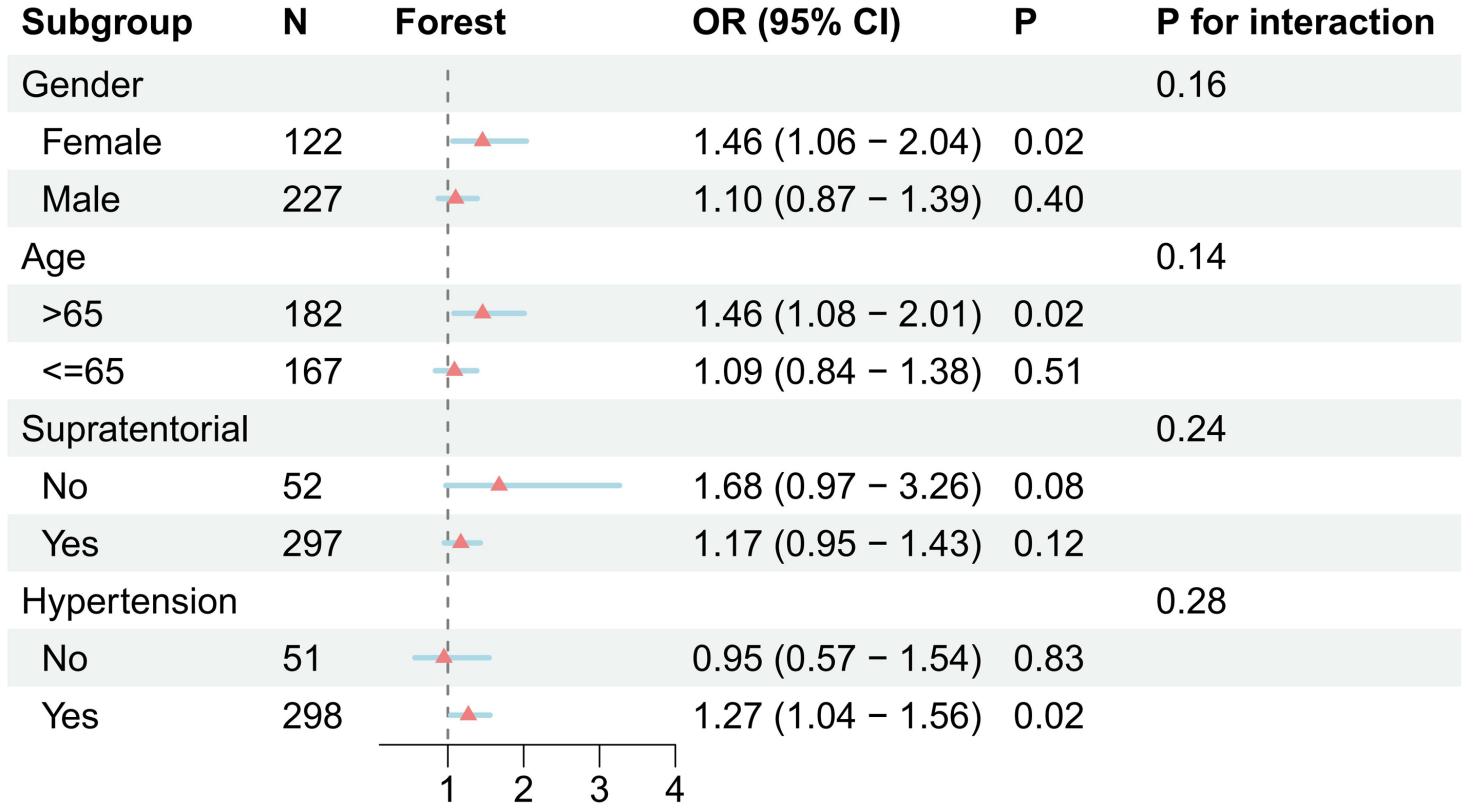
Subgroup analyses of the association between CONUT and HE OR, odds ratio; CI, confidence interval.

Subgroup analyses were conducted across clinically relevant strata including gender, age, hemorrhage location, and hypertension status. HE events numbered 29 in males versus 13 in females, 22 in patients aged ≥65 years versus 20 in those <65 years, 34 in supratentorial versus 8 in infratentorial hemorrhage, and 35 in patients with hypertension versus 7 without. Given the limited number of events in several strata, these subgroup analyses should be regarded as exploratory. The association between CONUT score and HE remained directionally consistent, with no statistically significant interactions detected (Figure 4, all *P* for interaction > 0.05).

Sensitivity analyses restricted to patients admitted within 24 h after symptom onset yielded comparable results (Table 3). When analyzed as a continuous variable, CONUT remained significantly associated with an increased risk of HE after full adjustment (Model 2: OR 1.32, 95% CI 1.05–1.64, *p* = 0.01). Patients in the highest CONUT quartile similarly demonstrated a significantly elevated risk compared with the lowest quartile. Component substitution sensitivity analyses were further performed by replacing CONUT with its individual components in otherwise identical multivariable models. These analyses did not identify a single component that accounted for the observed association of CONUT (Supplementary Table S3). A clinically anchored cut off sensitivity analysis was also conducted by dichotomizing CONUT as 0–4 versus ≥5. This analysis yielded consistent findings. Compared with CONUT 0–4, CONUT ≥5 was associated with a higher risk of HE in the fully adjusted model (Supplementary Table S4).

**Table 3:** Sensitivity analyses for the association between CONUT and HE.

Categories	Crude model OR (95% CI)	*P*	Model I OR (95% CI)	*P*	Model II OR (95% CI)	*P*
CONUT						
Continuous	1.24 (1.02–1.50)	0.02	1.23 (1.02–1.49)	0.03	1.32 (1.05–1.64)	0.01
Quartile 1 (*N* = 107)	Reference		Reference		Reference	
Quartile 2 (*N* = 85)	1.14 (0.44–2.96)	0.78	1.14 (0.43–2.97)	0.79	1.17 (0.44–3.11)	0.74
Quartile 3 (*N* = 69)	1.27 (0.47–3.40)	0.63	1.24 (0.45–3.39)	0.67	1.13 (0.39–3.23)	0.81
Quartile 4 (*N* = 54)	3.07 (1.24–7.57)	0.02	3.02 (1.21–7.53)	0.02	3.46 (1.13–9.15)	0.01

Model 1: adjusted for age and gender.

Model 2: adjusted for age, gender, hematoma location, onset-to-admission time, baseline hematoma volume, IVH, GCS and SBP.

CONUT: Quartile 1 (0–2), Quartile 2 (3–3), Quartile 3 (4–4), and Quartile 4 (5–10).

OR, odds ratio; CI, confidence interval; CONUT, controlling nutritional status; HE, hematoma expansion; GCS, glasgow coma scale; SBP, systolic blood pressure.

## 4 Discussion

In this retrospective cohort of patients with ICH, we evaluated the associations between three routinely available nutritional indices and HE. Among PNI, TCBI, and CONUT, only CONUT was positively associated with HE. This association remained consistent after adjustment and across sensitivity analyses. When categorized using a clinically anchored threshold, patients with CONUT ≥5 had a significantly higher risk of HE compared with those with scores 0–4, indicating that this cut-off identifies a higher-risk group at admission. In contrast, neither PNI nor TCBI showed significant associations after full adjustment. These findings support the clinical relevance of admission CONUT level, particularly values ≥5, in early risk stratification of acute ICH.

Although PNI, TCBI, and CONUT are all designed to assess nutritional status, their components differ in relevance to the biological processes underlying HE. HE is driven by early hemostatic instability and impaired vascular containment rather than by long-term nutritional reserve alone (22, 23). PNI is derived from serum albumin and lymphocyte count and primarily reflects protein reserve and immune status, but does not include lipid parameters involved in vascular structure and platelet function (11). In contrast, TCBI emphasizes energy reserves derived from serum triglycerides, total cholesterol, and body weight, but lacks indicators of protein availability and immune competence that are critical for acute hemostatic stabilization (24, 25). CONUT integrates albumin, lymphocyte count, and total cholesterol within a single score, allowing simultaneous assessment of protein support, immune status, and lipid-related vascular integrity (26). This broader compositional profile provides a plausible explanation for the more consistent and positive association observed between CONUT score and HE in the present study.

The association between CONUT score and HE reflects alterations in protein status, lipid-related vascular support, and immune regulation during the acute phase of ICH. Lower serum albumin levels indicate limited protein availability to maintain endothelial integrity and plasma oncotic pressure, thereby facilitating fluid extravasation and secondary bleeding at the hemorrhage site. Meanwhile, inadequate protein support limits the synthesis and stabilization of coagulation factors required for effective clot consolidation after initial hemostasis (27, 28). Reduced total cholesterol has been linked to structural vulnerability of the vascular wall, including medial smooth muscle cell degeneration, which lowers resistance to continued bleeding after vessel rupture. In addition, altered cholesterol content affects platelet membrane composition and activation pathways, attenuating platelet aggregation and compromising primary hemostasis (29–31). Lymphocyte count, another component of the CONUT score, reflects the inflammatory burden and immune suppression characteristic of the acute phase of intracerebral hemorrhage (32). Decreased lymphocyte levels have been implicated in coagulation imbalance, premature clot instability, and delayed resolution of proteolytic activity at the hemorrhage interface (33–35). Together, this integrated nutritional and immunological profile is associated with impaired early hemostatic stability and a higher risk of HE. Consistently, in our study, patients with HE exhibited prolonged activated partial thromboplastin time and reduced platelet counts at admission.

The incidence of HE in our cohort was 12.03%, lower than that reported in some ICH cohorts. HE incidence varies across studies and depends on follow-up imaging and outcome definitions, with reported rates ranging from 13 to 38% (5, 36). HE is strongly time dependent and occurs predominantly early after symptom onset (23, 37). Because HE adjudication required an assessable baseline and follow-up CT pair, exclusions related to the early clinical course may reduce the observed incidence. Patients hospitalized for <24 h, those undergoing hematoma evacuation before follow-up CT, and those with missing imaging or laboratory data are more likely to represent early deterioration, urgent surgery, early transfer, or early death. In these situations, serial pre-intervention imaging is less feasible, and HE cannot be adjudicated under an imaging-based definition. These requirements may shift the analyzable cohort toward patients stable enough to complete follow-up imaging, contributing to an HE incidence that differs from some prior cohorts. In this early, time-sensitive context, an admission-based and readily available marker may aid early risk stratification. In our study, CONUT was consistently associated with HE in the overall cohort. Similar patterns were observed in sensitivity analyses restricted to patients admitted within 24 h after symptom onset. These findings support the relevance of admission-based nutritional risk assessment of HE in ICH.

Although the findings are encouraging, several limitations should be acknowledged. First, due to the retrospective design, certain imaging features such as the CTA spot sign and non-contrast CT markers (e.g., blend, black hole, and island signs) were not consistently recorded in a structured format and therefore could not be reliably retrieved for analysis. Second, the single-center retrospective nature of the study limits generalizability and precludes causal inference. Third, nutritional status was assessed only at admission without evaluation of dynamic changes during hospitalization. Fourth, although major confounders were adjusted for, residual confounding related to early management strategies, including blood pressure control and hemostatic therapy, cannot be fully excluded. Fifth, the onset-to-follow-up CT interval was not standardized, and residual bias related to heterogeneous timing cannot be completely eliminated. Finally, the limited number of HE events may have reduced statistical power in subgroup analyses. Future multicenter prospective studies are needed to validate these findings. Standardized imaging protocols, predefined onset-to-follow-up CT intervals, longitudinal nutritional assessment, and more detailed treatment data would strengthen the robustness and clinical applicability of the results.

## 5 Conclusion

In conclusion, among the nutritional indices evaluated, only the CONUT score was associated with HE in patients with ICH. As a simple nutritional risk screening tool assessed at hospital admission, CONUT may help identify patients with acute nutritional and metabolic vulnerability who are at higher risk of early hematoma progression. These findings highlight the potential value of early nutritional risk assessment in ICH and warrant further prospective studies to clarify the role of nutritional status in the pathophysiology of HE.

## Data Availability

The original contributions presented in the study are included in the article/Supplementary material, further inquiries can be directed to the corresponding author.
